# Numerical Approach to Modeling and Characterization of Refractive Index Changes for a Long-Period Fiber Grating Fabricated by Femtosecond Laser

**DOI:** 10.3390/ma9110941

**Published:** 2016-11-21

**Authors:** Akram Saad, Yonghyun Cho, Farid Ahmed, Martin Byung-Guk Jun

**Affiliations:** 1 Department of Mechanical Engineering, University of Victoria, Victoria, BC V8W 2Y2, Canada; akrams@uvic.ca (A.S.); yhc81@uvic.ca (Y.C.); fahmed@uvic.ca (F.A.); 2 School of Mechanical Engineering, Purdue University, West Lafayette, IN 47906, USA

**Keywords:** refractive index, Gaussian beam, femtosecond laser, LPFG model, beam envelop, grating period, grating length, index sensor

## Abstract

A 3D finite element model constructed to predict the intensity-dependent refractive index profile induced by femtosecond laser radiation is presented. A fiber core irradiated by a pulsed laser is modeled as a cylinder subject to predefined boundary conditions using COMSOL5.2 Multiphysics commercial package. The numerically obtained refractive index change is used to numerically design and experimentally fabricate long-period fiber grating (LPFG) in pure silica core single-mode fiber employing identical laser conditions. To reduce the high computational requirements, the beam envelope method approach is utilized in the aforementioned numerical models. The number of periods, grating length, and grating period considered in this work are numerically quantified. The numerically obtained spectral growth of the modeled LPFG seems to be consistent with the transmission of the experimentally fabricated LPFG single mode fiber. The sensing capabilities of the modeled LPFG are tested by varying the refractive index of the surrounding medium. The numerically obtained spectrum corresponding to the varied refractive index shows good agreement with the experimental findings.

## 1. Introduction

Long-period fiber gratings (LPFGs) are fiber optic devices with various applications in optical communications and sensing systems [1]. Fiber gratings are produced by periodically altering refractive index inside the core region of an optical fiber [2]. Several techniques are available to produce LPFGs, such as those based on ultraviolet, CO_2_, and femtosecond laser radiation, with the latter being the most versatile method [1,3,4,5]. LPFGs are usually fabricated with grating periods on the order of 100 micrometers to a millimeter. LPFGs are characterized by coupling light from a guided core mode into forward propagating cladding modes [5]. LPFGs are currently considered to be simple yet versatile devices for various sensing applications [6]. In-fiber long-period gratings have been demonstrated as effective and efficient tools for refractive index sensing [5,7].

Due to their ability to produce focused pulses, femtosecond laser systems have been recently used to fabricate LPFGs offering permanent refractive index increase in various glasses [8,9,10,11]. In such systems, light intensity is highly localized at the focal point resulting in inducing periodical refractive index modulation in the core of fiber without significantly affecting the cladding or the polymer coatings. LPFGs fabricated by femtosecond laser have superior aging characteristics and high resistance to thermal decay [9,11]. For effective fabrication, it is crucial to understand and model the fabrication process and characterize the index changes by femtosecond laser. The basic physical characteristic of optical waveguides is given by the distribution of the induced refractive index. From this distribution, all local parameters can be computed. Consequently, measurement of the refractive-index profiles provides valuable quality control during the development and production of the fiber optic components. Several technical approaches are reported to measure the refractive index profile of optical fibers [12,13,14,15,16,17,18]. However, the experimentally based approaches involve time-consuming cycles of design. Thus, computational methods and computerized modeling play a vital role in reducing the time and its associated costs as well as investigating novel phenomena that may not lend themselves to experimental procedures [19]. Mathematical models, the phase grating and Gaussian grating, have been extensively used in estimating the refractive index profile. However, the effect of the self-focusing is not explicitly reported in these models [20,21].

For design and fabrication purposes, self-focusing is of importance, as the modification of the beam must be taken into consideration [22]. The beam propagation method [23,24], nonlinear Schrödinger equation [25,26,27], and finite-difference time-domain method [28,29] have been employed to understand the temporal and spatial electromagnetic field variations in nonlinear bulk media including the self-focusing effect. By using a non-paraxial beam propagation method based on the Helmholtz equation, a non-paraxial treatment of the nonlinear process is reported to limit the reduction in beam size to the order of one optical wavelength, leading to multiple foci, beam breakup, and filament formation [23]. The multiple filamentation is reported to be suppressed for the circularly polarized beams in a Kerr medium using a scalar equation to describe self-focusing in the presence of vectorial and non-paraxial effects [26]. The finite-difference time-domain method has also been utilized to study the complex nonlinear phenomenon [28,29]. Given the rapid development of the computer industry, the finite element method has become an indispensable computation tool for solving engineering problems in electromagnetism. This numerical simulation method is established on the basis of variational principles, domain meshing, and interpolation function [30,31]. The refractive index profile induced by CO_2_ laser was calculated using an empirical formula based on numerically obtained thermal stresses [32]. A multi-layer thin lens self-focusing model that considers the refractive index and diffraction effects was proposed to analyze the self-focusing behavior of light beams [33]. The reported methods rely on mathematical functions to describe the refractive index profile independently of the laser intensity distribution within the focal volume [23,24,25,26,27,34]. Thus, with proper definition of the boundary conditions and full incorporation of the laser intensity field the finite element method can be utilized to account for the refractive index perturbation due to femtosecond laser. In this work, a finite element based approach that accounts for the refractive index dependency on femtosecond laser intensity is proposed. The robustness of the model is achieved by implementing the Beam Envelope Method to overcome the demanding computation criteria.

In addition to the significance of characterizing the induced refractive index, recent advances in the field of guided-wave optics such as fiber optics have prompted the introduction of waveguide numerical modeling [35,36]. The most widely used numerical methods for photonics devices include Galerkin and moment methods, transfer matrix method, finite difference based methods, transmission line matrix methods, stochastic methods such as the Monte Carlo method, and finite element-based methods [9,37]. A general theoretical method for the transmittance calculation of ultraviolet-written gratings is reported [20]. The method is based on the coupled mode theory in step-index optical-fibers. The interactions between core mode and cladding modes in a cross-section of an LPFG are reported using an approach based on the coupled mode theory and transfer-matrix methods [11]. An empirical relation accounting for the induced refractive index for a uniform pure silica fiber experiencing uniaxial tension is reported [38]. In other work, the refractive index profile induced by CO_2_ laser is calculated using the aforementioned empirical formula based on numerically obtained thermal stresses [32]. An integration of a perfectly matched layer, perfectly reflecting the boundary, object meshing method, and boundary meshing method into the finite element model and eigenmode expansion method is presented to correlate the design period to the spectral characteristics of superstructure fiber Bragg gratings [21]. Most of the reported FEM models solely consider a cross-section of the waveguide in the analysis to avoid the daunting computation requirements [19,21,32,36,39,40,41,42]. Long-period fiber grating devices in particular are characterized by their large sizes compared to the wavelength of the guided light. Moreover, the reported methods lack explicit coupling between the numerically induced refractive index profiles with the proposed numerical models. In this work, a 2D finite element based model constructed in the COMSOL 5.2 Wave Optics Module is coupled with the proposed refractive index model to fully describe the propagation of light through a long period grating single mode fiber in the frequency domain. The demanding computation requirements are overcome by using the beam envelope method. Additionally, the constructed model is tested as a refractive index sensor.

## 2. Intensity-Dependent Refractive Index Model

Several optical waveguides and devices operate in a regime where the dielectric polarization of the material responds linearly to the electric filed of the light and the refractive index is independent of the intensity of optical beams. However, in the nonlinear regime the dielectric response of the material is nonlinear and the material refractive index is dependent on the optical filed intensity. Thus, the optical properties, behavior, and performance of the device become dependent on the optical beam intensity [43,44,45]. As the intense laser beam hits the core, the generated electric field perturbs the electron clouds around the nuclei leading to a significant refractive index dependency on laser intensity [46,47]. With progressive intensification of the laser power, noise across the beam’s cross-sectional intensity distribution can be amplified, resulting in the beam breaking up into several self-focused filaments. The dielectric exhibits pulse intensity dependent index of refraction that is given by [48,49]:

(1) $$n=n_{o}+\gamma I$$

where *n_o_* is the nominal refractive index, *γ* is the nonlinear refractive index coefficient, and *I* is the laser intensity.

**Figure 1:**
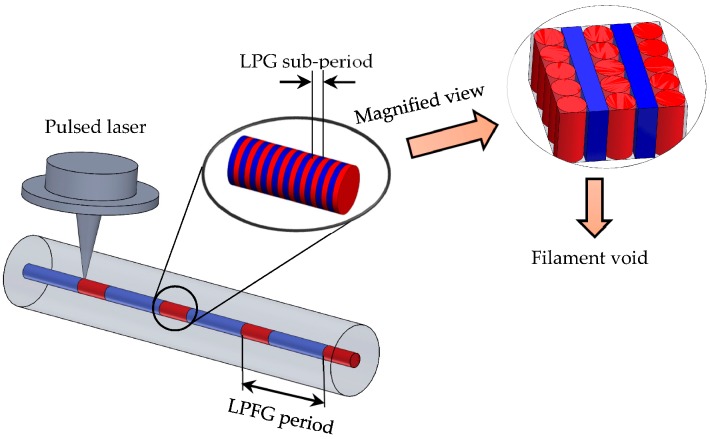
Schematic illustration of long-period grating fabrication.

The proposed numerical approach is carried out by implementing a 3D finite element model to predict the refractive index profile induced by femtosecond laser irradiation. A fiber core irradiated by a single shot of a pulsed laser can be modeled as a cylinder subject to predefined boundary conditions using the Wave Optics Module built in COMSOL5.2 Multiphysics [47,50]. Based on the fabrication mechanism shown in Figure 1, the generated refractive index profile within a cylinder is of a major interest.

The commercial single mode fiber used in this work is Corning SMF-28 (New York, NY, USA). The information provided by the manufacturer indicates that the effective refractive index of the fiber is 1.4620 for a wavelength equal to 1550 nm. It is also mentioned in the specification sheet that the refractive index of the core is 0.36% higher than that of the cladding. To determine the nominal refractive indices of the core and the cladding, a mode analysis is carried on a cross-section of the fiber. In this analysis, the concept of reverse engineering is to be used, which means that the refractive index of the core is to be parametrically swept to obtain a discrete effective index for each value of the sweep. An eigenvalue equation for the electric field *E* is derived from Helmholtz equation and solved for the eigenvalue *λ* = −*jβ* [50,51].

(2) $$\nabla \times \left(\nabla \times E\right)-\frac{\omega^{2}}{c_{o}^{2}}n^{2}E=0$$

where *E* is the electric field, *c_o_* is the speed of light, *n* is the refractive index, *ω* is the angular frequency, function and *β* is the propagation constant.

**Figure 2:**
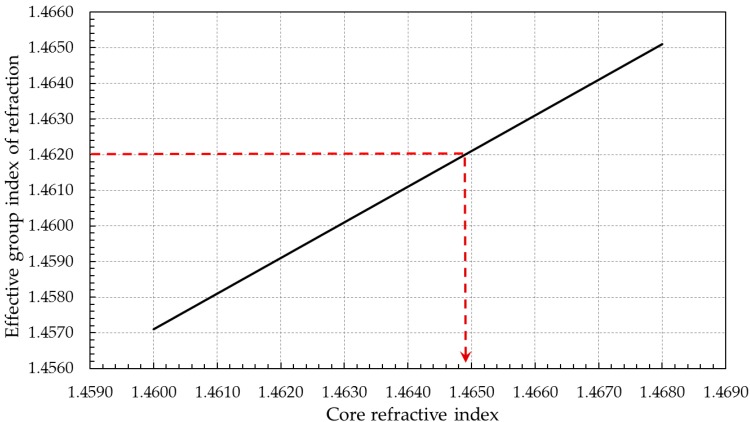
The numerical effective refractive index of the SMF.

**Figure 3:**
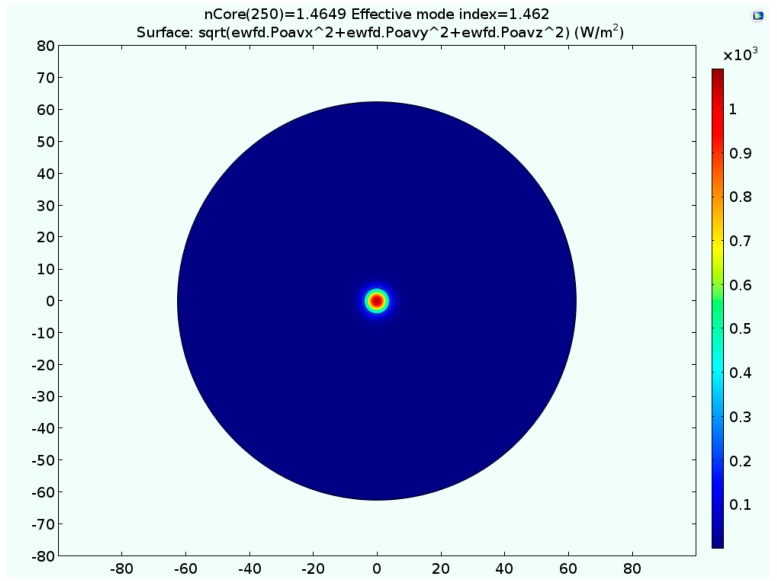
Visualization of the light intensity in the cross section of the SMF; the core’s index is 1.4649.

Since the amplitude of the electric field decays rapidly as a function of the radius of the cladding, the electric field is set to zero on the cladding’s periphery. Figure 2 shows the effective refractive index of the fiber as a function of the swept refractive index of the core. The obtained results indicate that a value of the core’s refractive index equals to 1.4649 corresponds to a targeted effective index having a value of 1.4620. The confinement of the light in a single mode fiber having the aforementioned refractive indices is shown in a surface plot depicted in Figure 3. The obtained refractive indices of both the core and the cladding are to be used in the intensity-dependent refractive index model.

The proposed intensity-dependent refractive index model in this work is an extension of the 3D model presented in reference [50] to account for femtosecond laser fabrication. The modeling scheme is carried out using the Beam Envelope method. As the electric field envelope has a slower spatial variation than the electric field, a coarse mesh can be used. Since the laser beam propagates essentially in one direction, it is assumed that the electric field, *E*, of the wave can be written as [50]:

(3) $$E=E_{1}e^{-j\beta x}$$

where *E*_1_ is a slowly varying field envelope function. Inserting this electric field formulation into the Maxwell’s equations results in the following wave equation for the envelope function [47,50]:

(4) $$\left(\nabla -j\beta \right)\times u_{r}^{-1}\left(\left(\nabla -j\beta \right)\times E_{1}\right)-\frac{\omega^{2}n^{2}}{c_{0}^{2}}E_{1}=0$$

where *μ_r_* is the relative permeability, and *c*_0_ is the speed of light.

**Figure 4:**
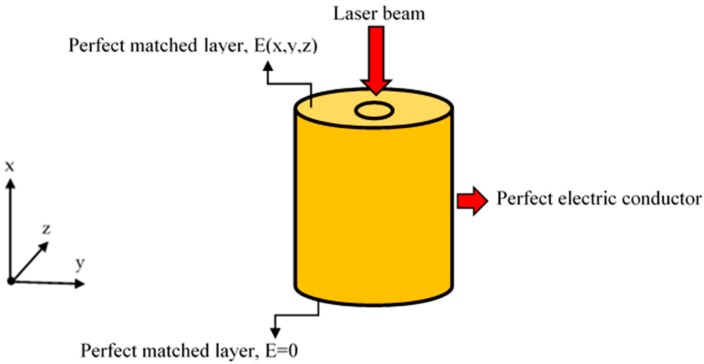
Schematic illustration of the model geometry and boundary conditions.

As shown in Figure 4, the cylinder is subject to an incident beam approximated by a Gaussian beam propagating in the x-direction and polarizing in the z-direction. To absorb the incident radiation without producing reflections, a perfect matched layer (PML) boundary conditions are applied to the top and bottom of the cylinder to account for the electric filed distribution of the incident Gaussian beam [19,20]. In addition to the PML, the periphery of the cylinder is considered as a perfect electric conductor. For the model considered in this work, the beam waist, cylinder height, laser energy, laser repetition rate, pulse width are set to be 0.50 μm, 8.30 μm, 0.50 μJ, 1 kHz, and 120 fs respectively. The top surface of the cylinder is meshed using a free triangular meshing pattern. The generated mesh surface is then swept across the entire model. Using the beam envelope method, it is found that the solution is mesh-independent for values of the maximum element size of the top surface and the maximum mesh size of the swept mesh to be one third of the laser wavelength (*λ*) and the relight range(*x*_0_), respectively.

**Figure 5:**
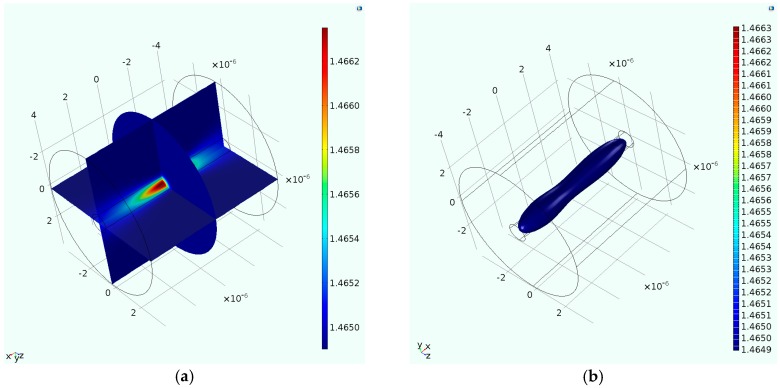
(**a**) A multi-slice plot of the induced refractive index profile; (**b**) An isosurface plot illustrating specified contours of the induced refractive index.

The surface plot of the refractive index profile (RIP) is illustrated in the multi-slice plot shown in Figure 5a. The averaged refractive index over the entire inner cylinder for the conditions considered in this work is 1.4655 demonstrating a refractive index change of 6 × 10^−4^. To further explore the growth of the induced refractive index profile, an isosurface plot with refractive indices ranging from the nominal value- 1.4649- to the maximum refractive index value- 1.4663- is shown in Figure 5b. As shown in the figure, averaging the refractive index within the inner cylinder seems to provide reliable characterization of the induced refractive index. It should be noted that the proposed model is limited to predict refractive index profiles induced by peak powers that are lesser than three times of the critical power. This limitation is deemed to be a result of not accounting for the effect of the multi-photon ionization. The latter phenomenon has been reported to be pronounced for high laser powers [52]. The induced refractive index change due to the simulated fabrication conditions is to be implemented in the modeling scheme of the LPFG sensor.

## 3. Long-Period Grating Model

**Figure 6:**
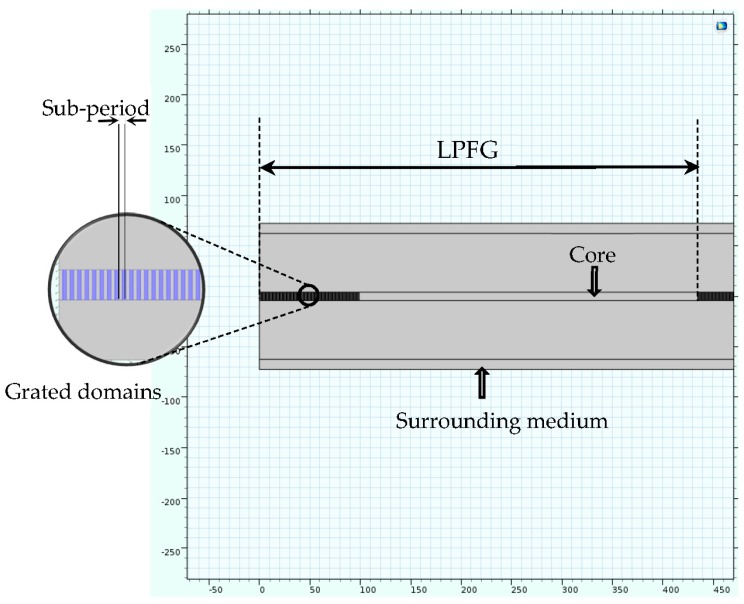
Geometrical depiction of the numerically modeled single mode fiber.

The geometry of the 2D model is illustrated in Figure 6. The in-fiber fabricated regions of the pure silica single-mode fiber are dealt with as rectangles with refractive indices obtained from the refractive index model. This model considers a section of a dielectric slab waveguide that is finite in the x and y directions. Because the fields drop off exponentially outside the waveguide, the fields can be assumed to be zero close to the periphery. Thus, the air boundary is assumed to be subject to the perfect electric conductor boundary condition.

**Figure 7:**
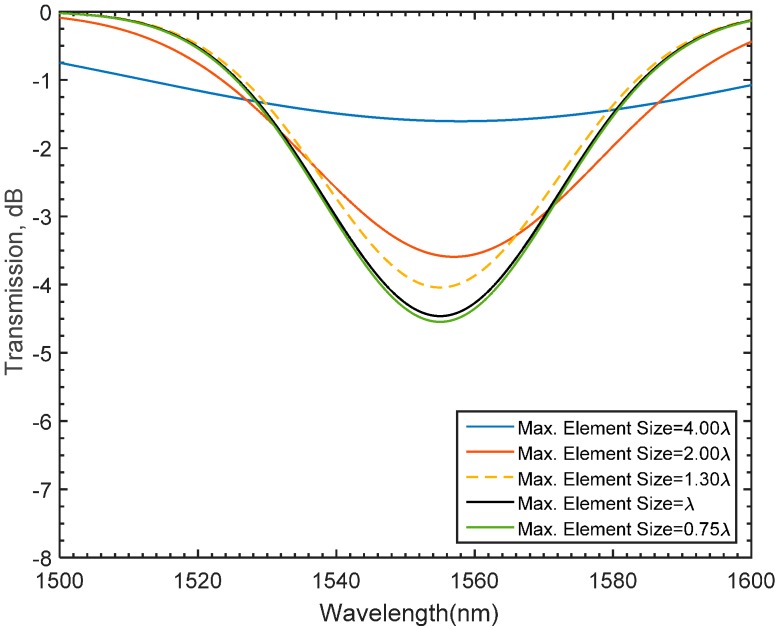
Gradual growth of LPFG in the modeled single mode fiber for different number of periods using free triangular mesh.

**Figure 8:**
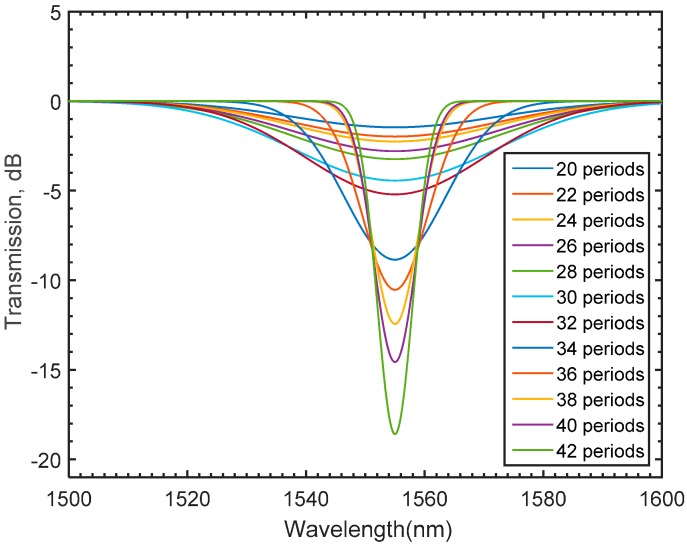
Gradual growth of LPFG in the modeled single mode fiber for different number of periods using mapped mesh.

The numerical port boundary conditions in the x direction are utilized to model the guided wave propagating in the x direction and calculate the generated spectrum. The generated electric filed is obtained by solving for the beam envelope. The Electromagnetic Waves, Beam Envelopes physics interface is used to handle the propagation over distances that are many wavelengths long. Since the wave propagates essentially in one direction, the propagation constant in the x-direction can be assigned to the modeled regions [47,50]. Based on the nature of the beam envelope method, the meshing requirements of the LPFG model are expected to be computationally inexpensive, which means that coarse meshing can be utilized. Thus, a mesh convergence study is essential for the numerically obtained solution to be mesh independent. Accordingly, a simulation study of a long period fiber grating for various second order Lagrangian mesh element sizes is carried out. The number of periods, grating length, and grating period are set to be 30, 100, and 435 μm, respectively, while the maximum element size is varied. The spectra of the assigned mesh sizes are superimposed in Figure 7. The plotted results suggest that a maximum mesh size equal to the value of the wavelength is adequate for the simulated results to be mesh-independent. Consequently, one might notice that a significant reduction in the meshing requirements has been achieved confirming that the beam envelope method can substantially alleviate the daunting computational demands that solving for the full wave method would normally require. To investigate the spectral growth resulted from varying the number of grating periods (*α*), various simulation studies are conducted. In these studies, both the grating length and grating period are kept constant to be 100 μm, and 435 μm respectively. As shown in Figure 8, the spectral growth of an LPFG seems to evolve for various numbers of the grating periods around a relatively constant attention dip, 1555 nm. To fully verify whether the numerically obtained results using the BEM provide reliable numerical interpretation of the simulated spectrum, a set of experiments with identical fabrication conditions are carried out.

## 4. Spectral Growth Experimental Validation

**Figure 9:**
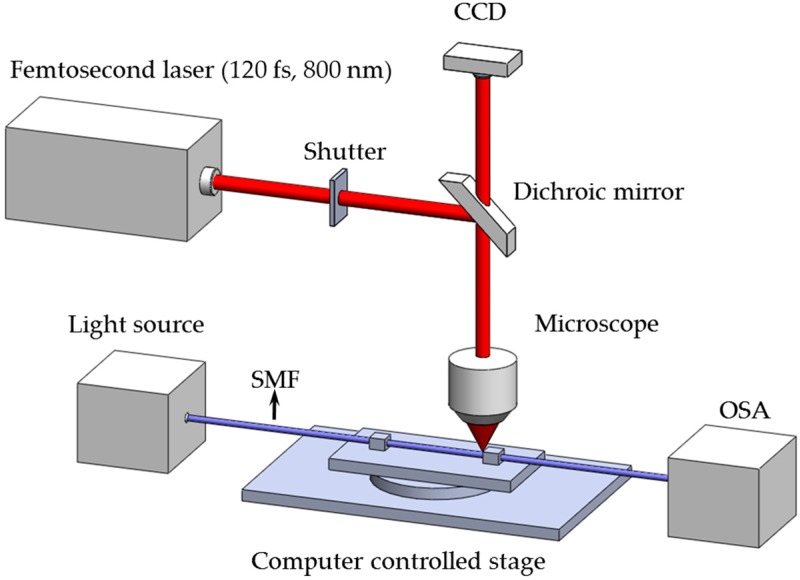
Schematic illustration of the experimental setup.

In this section, the numerically obtained spectra are to be experimentally verified. The refractive index change in the fiber core was carried out using femtosecond laser (Spectra-Physics ultrafast Ti: Sapphire laser, Spectra-Physics, Santa Clara, CA, USA) with a pulse width of 120 femtosecond and a center wavelength of 800 nm. The optical fiber was firmly mounted on a computer-controlled 3-axis stage for alignment with submicron precision. The laser beam was reflected by a dichroic mirror and delivered to the objective lens (Numerical aperture: 0.55) and then focused into the fiber core. The LPFG fabrication process can be monitored by the CCD camera above the dichroic mirror. The period and length of refractive index modulation of the fabricated LPFGs are 435 μm and 100 μm respectively. The fiber was coupled with a broad-band light source (AFC BBS-1550) and a spectrum analyzer (PHOTONETICS Walics, PHOTONETICS INC., Peabody, MA, USA) to monitor the growth of LPFG during fabrication. A schematic illustration of the experimental setup is shown in Figure 9.

**Figure 10:**
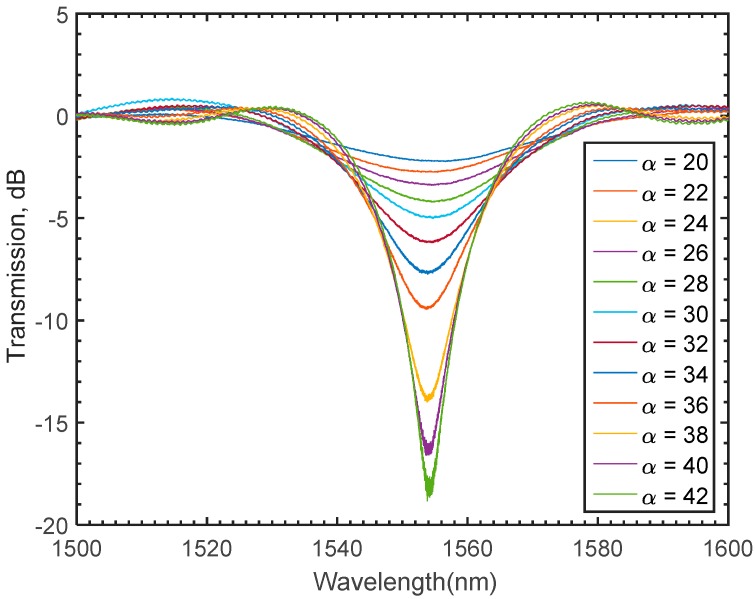
Gradual growth of LPFG in SMF fiber for different number of periods.

The spectral growth of the inscribed fiber with an attenuation dip of 19 dB at 1555 nm is shown in Figure 10. The results plotted in Figure 8 and Figure 10 clearly state that a good agreement between the numerically obtained results and the experimental findings is achieved. Thus, the beam envelope technique is to be implemented in this work to numerically design an LPFG refractive index sensor. However, full numerical design of the proposed model necessitates further investigation of the proper selection of the grating period and length.

## 5. Varying the Grating Length and Grating Period

**Figure 11:**
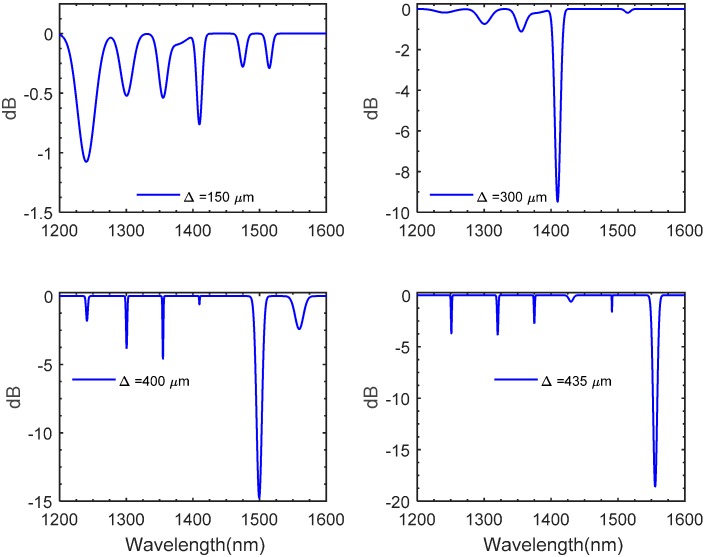
Transmission spectrum of an LPFG numerically modeled in SMF with various grating periods; the number of grating periods and the grating length are 42 and 100 μm respectively.

**Figure 12:**
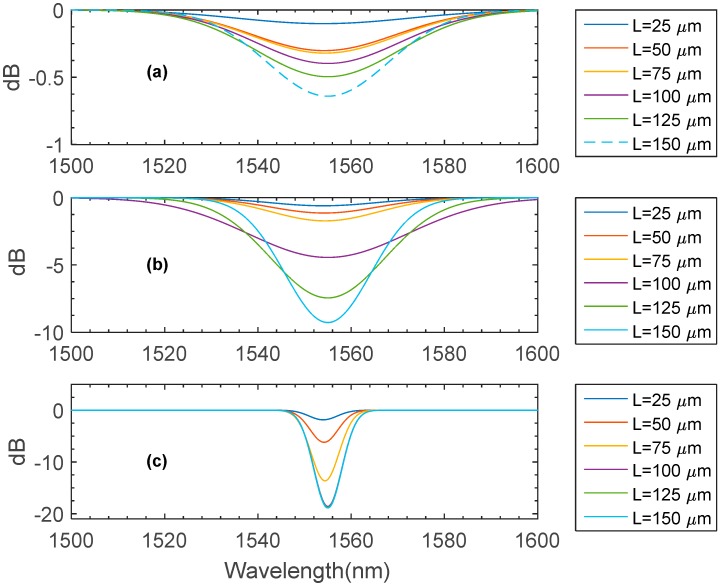
The numerically acquired spectrum for various grating lengths; the grating period is 435 μm. The numbers of periods in (**a**–**c**) are 15, 30, and 42 respectively.

As the fabrication of an LPFG sensor chiefly relies upon the introduction of a periodic modulation mainly achieved by permanent modification of the refractive index of the core, characterizing the fabrication variables such as the grating period (***Δ***), and grating length (***L***) are of a major importance to reliably design and fabricate LPFG sensors. The effect of varying the grating period of the modeled LPFG in this work is illustrated in Figure 11. The plotted results clearly show that for each period there is a unique resonant wavelength. The numerical results indicate that as the gating period increases, the resonant curve tends to get narrower and deeper, suggesting that a grating period equal to 435 μm is an optimal choice to potentially increase the sensing sensitivity. The effect of numerically assigning different grating lengths is shown in Figure 12. In this plot, three sets of numerical studies are carried with assigned number of grating periods of 15, 30, and 45 periods while the grating period, ***Δ***, is fixed at a value of 435 μm. As stated in Figure 12a,b, setting the number of periods to be 15 and 30 seems to provide wider and shorter attention dips. The plotted results shown in Figure 12c suggest that fabricating the LPFG model with 42 periods causes the attention dips to rapidly surge, forming narrower valleys as the grating length reaches a value of 100 μm, whereas further increments in the grating length seem to have an insignificant impact on the spectrum. Thus, in this work, the values of the grating period, grating length, and number of periods are 435 μm, 100 μm, and 42 periods respectively.

## 6. Experimental Validation of the Modeled Refractive Index LPFG Sensor

In this section, the sensing principles of an LPFG refractive index sensor are to be numerically presented and experimentally validated. Due to the coupling of fiber optic core modes into the cladding mode, the transmission valley appears. The phase matching between forward-propagating core mode and cladding mode in an LPFG is governed by [6,18]:

(5) $$\lambda_{res}=\left[n_{eff}^{core}\left(\lambda_{res}\right)-n_{eff}^{cl\left(m\right)}\left(\lambda_{res}\right)\right]∆$$

where *λ_res_* is the resonant wavelength, $n_{eff}^{core}$ is the effective index of the core mode, and $n_{eff}^{cl\left(m\right)}$ is the effective index of the mth-order cladding mode.

**Figure 13:**
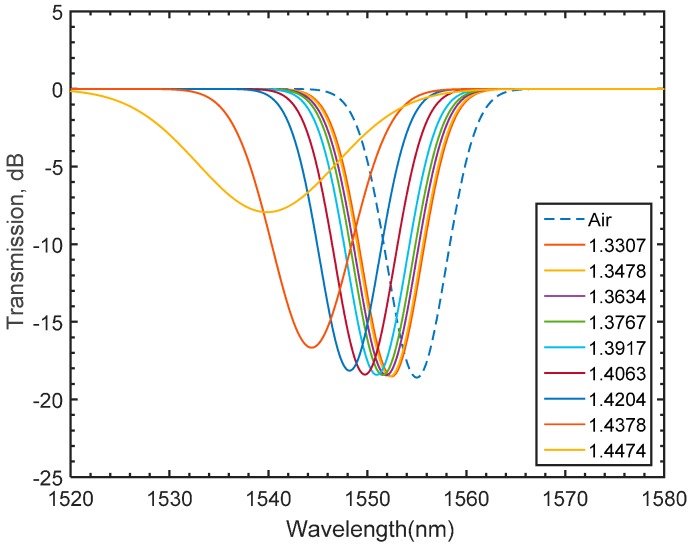
The numerically obtained spectral growth as a function of medium refractive index.

**Figure 14:**
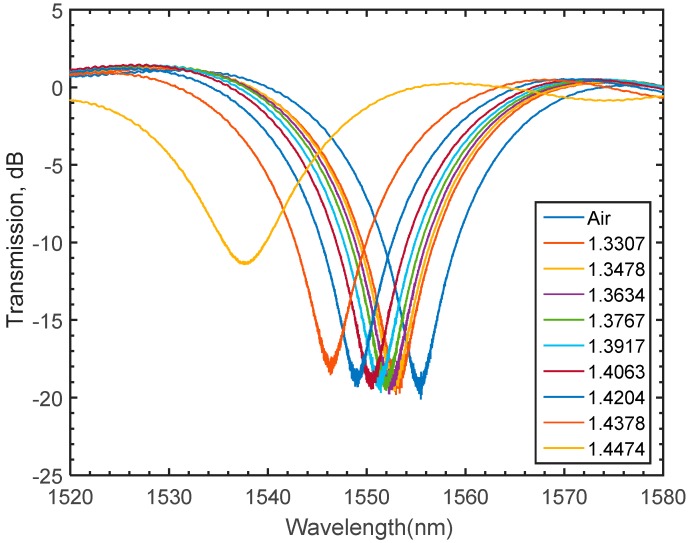
Experimentally obtained shift of the transmission valley when the LPFG is exposed to solutions of different refractive indices.

**Figure 15:**
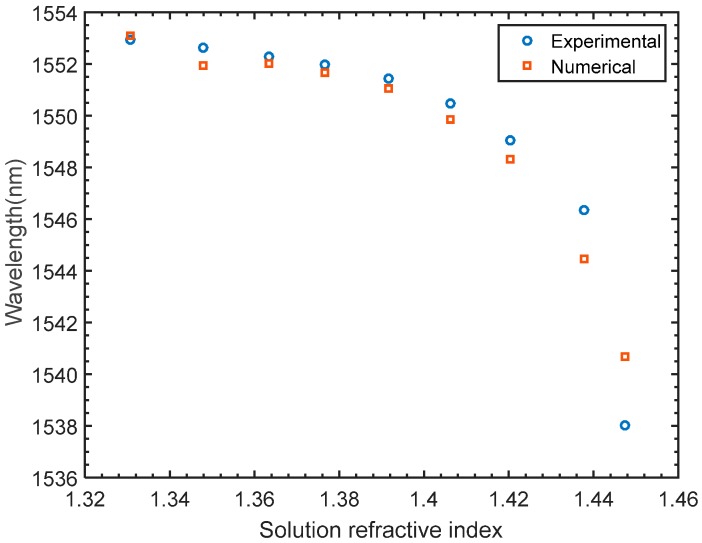
Numerical vs. experimental refractive index characterization of an LPFG sensor.

Due to absorption and scatterings, the cladding modes tend to escape from the fiber into the surrounding medium making LPFGs superior refractive index sensing tools. As stated in Equation (5), the resonant wavelength is a function of the effective indices of the core and cladding modes as well as the grating period [6,18,20]. As the effective indices of the cladding modes depend on the ambient, a change in the ambient index is likely to shift the value of resonant wavelength. In the proposed numerical model, the variation of the refractive index of the medium is achieved by conducting a parametric sweep of the refractive index values on the regions enclosing the SMF domains. The numerical results are plotted in Figure 13, showing a left shift of the transmission spectrum as the value of the swept refractive index increases. To verify the numerical variations of the refractive index of the medium on the light spectrum, sets of experiments are carried out at room temperature. In each set of the experiments, the fabricated grating is immersed in a glycerin solution. The gradual variation of the glycerin concentration is set such that the refractive index varies from 1.3307 to 1.4474. The spectral behaviour of varying the refractive index of the solution is captured and recorded using the spectrum analyzer. The effect of varying the refractive index of the medium on the spectrum for the LPFG inscribed in pure silica core SMF is shown in Figure 14, where a shift of the attention dip to the left gradually occurs as the index increases. As shown in Figure 15, the numerical and experimental spectral shift of the transmission valley increases non-linearly as the medium refractive index increases. For the refractive indices tested in this work, a maximum deviation between the numerical and experimental is found to be in the order of 0.175%, which confirms adequate agreement between the numerical and experimental findings.

## 7. Conclusions

Two finite element models constructed in the COMSOL5.2 Wave Optics Module and correlating the induced refractive index profile generated by a femtosecond laser inscription to an LPFG model of a single-mode fiber are presented. The demanding computational requirements of the proposed FE models are overcome by using the beam envelope method. The first model is a 3D model and is capable of characterizing the intensity-dependent refractive index of the core. The induced refractive index is deployed in an LPFG model portrayed as a 2D dielectric slab. The LPFG model simulates the spectral distribution generated from the interaction between the light and the SMF. The analysis of the simulation results conducted in this work suggests that the values of the number of grating periods, grating period, and grating length are 42, 435, and 100 μm, respectively. The spectral growth of the LPFG model resulting from setting the averaged laser energy to be 0.50 μJ for various numbers of periods is experimentally validated using identical inscribing conditions. The numerically obtained results exhibit good agreement with the experimental data. Additionally, the refractive index of the domains surrounding the modeled SMF is varied to account for various glycerin solution concentrations. Non-linear shifts of the numerically acquired spectra for various refractive indices are observed and successfully verified by experimentally immersing the gratings in a glycerin container.
